# Recent Genome-Editing Approaches toward Post-Implanted Fetuses in Mice

**DOI:** 10.3390/biotech12020037

**Published:** 2023-05-11

**Authors:** Shingo Nakamura, Emi Inada, Issei Saitoh, Masahiro Sato

**Affiliations:** 1 Division of Biomedical Engineering, National Defense Medical College Research Institute, Saitama 359-8513, Japan; 2 Department of Pediatric Dentistry, Graduate School of Medical and Dental Sciences, Kagoshima University, Kagoshima 890-8544, Japan; 3 Department of Pediatric Dentistry, Asahi University School of Dentistry, Mizuho-shi 501-0296, Japan; 4 Department of Genome Medicine, National Center for Child Health and Development, Tokyo 157-8535, Japan

**Keywords:** in utero gene delivery, genome editing, electroporation, fetuses, CRISPR/Cas9, tail-vein injection, transplacental gene delivery, knock-in, knock-out, indels

## Abstract

Genome editing, as exemplified by the CRISPR/Cas9 system, has recently been employed to effectively generate genetically modified animals and cells for the purpose of gene function analysis and disease model creation. There are at least four ways to induce genome editing in individuals: the first is to perform genome editing at the early preimplantation stage, such as fertilized eggs (zygotes), for the creation of whole genetically modified animals; the second is at post-implanted stages, as exemplified by the mid-gestational stages (E9 to E15), for targeting specific cell populations through in utero injection of viral vectors carrying genome-editing components or that of nonviral vectors carrying genome-editing components and subsequent in utero electroporation; the third is at the mid-gestational stages, as exemplified by tail-vein injection of genome-editing components into the pregnant females through which the genome-editing components can be transmitted to fetal cells via a placenta-blood barrier; and the last is at the newborn or adult stage, as exemplified by facial or tail-vein injection of genome-editing components. Here, we focus on the second and third approaches and will review the latest techniques for various methods concerning gene editing in developing fetuses.

## 1. Introduction

Genome editing, as exemplified by clustered regularly interspaced short palindromic repeats (CRISPR)/CRISPR-associated protein 9 (Cas9) system, has been widely recognized as a useful tool for various biological studies, including analysis of gene function, creation of animal disease models, and improvement of genetically transformed organisms. CRISPR/Cas9-mediated gene knock-in (KI) or knockout (KO) has been extensively applied to cells derived from various species and organisms, including plants, fish, amphibians, and mammals (reviewed by Harrison et al. [1] and Hsu et al. [2]). In bacteria, the CRISPR/Cas9 system produces a specific double-strand break (DSB), which is successfully achieved by specific double-stranded DNA (dsDNA) cleavage after binding a bacterial-derived nuclease (such as Cas9) complexed with a short guide RNA (gRNA) to a specific chromosomal locus. This event (specific DSB) will be recovered through the cell’s DNA repair machinery, where the two edges are rejoined via either non-homologous end joining (NHEJ) or through homology-directed repair (HDR). The former is achieved by relegating the broken ends in the absence of a DNA donor and often causes alteration of nucleotides nearby the DSB site, leading to random insertions or deletions of nucleotides, called “indels”. The latter is achieved by relegating the broken ends to the presence of an appropriate DNA donor and relies on the availability of homologous regions of the donor DNA used. Therefore, the HDR-mediated genome editing efficiency is generally lower than that of the NHEJ-based one. Furthermore, HDR preferentially occurs in dividing cells, whereas NHEJ occurs in both dividing and nondividing cells [3].

**Figure 1:**
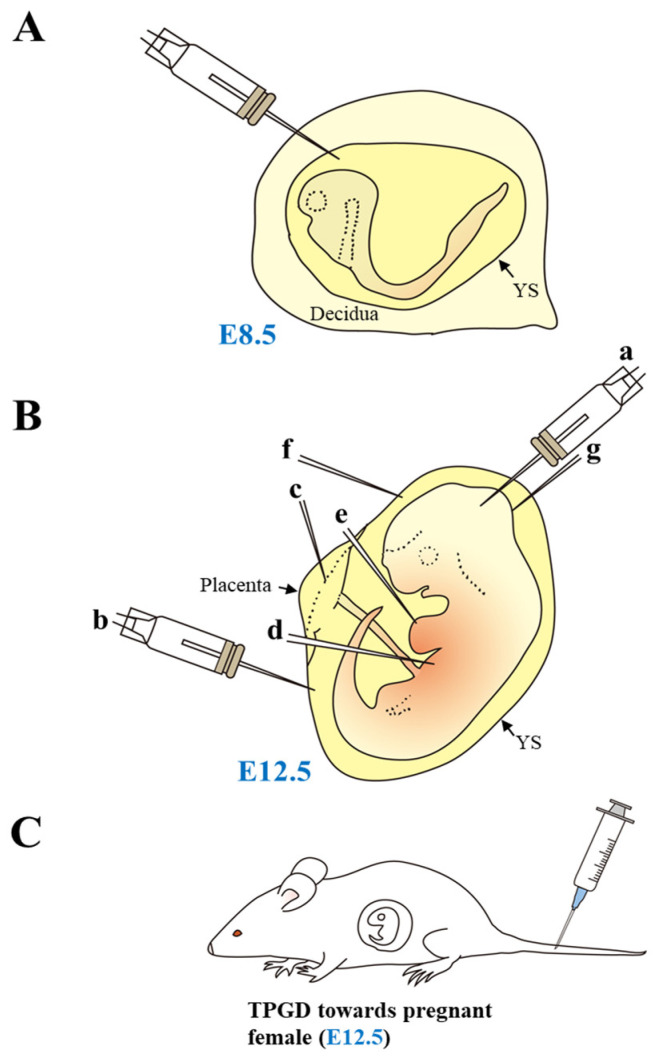
Gene delivery into early-to-mid-gestational fetuses. (**A**) Gene delivery into somite-stage embryos. Introduction of exogenous nucleic acids into the abdomen of anesthetized pregnant female is possible through a glass micropipette under a dissecting microscope after exposure of the uterus. (**B**) Gene delivery into fetuses at embryonic day (E) 12.5. At E12.5, the fetus (embryo) is visible through the yolk sac (YS) upon surgical dissection of the uterus under a dissecting microscope. Thus, it is possible to administer intrabrain (a), intraamniotic (b), intraplacental (c), intrahepatic (d), intracardiac (e), intravitelline (f), and skin (g) injections of genome-editing components using a micropipette for in utero gene delivery. (**C**) Gene delivery into fetuses using transplacental gene delivery (TPGD). Tail-vein injection of a solution containing nucleic acids into pregnant female mice is also a useful in vivo approach to introduce nucleic acids into E9.5–12.5 fetuses. This figure was drawn in-house and reproduced with permission from Sato et al. [6], published by MDPI, 2020.

In the case of genome editing in mammals, at least four genome-editing approaches have been reported. The first approach, referred to as “germline editing”, involves genome editing of germline cells (primordial germ cells, gamete progenitors, gametes, fertilized eggs [zygotes], and preimplantation stage embryos) to obtain gene-engineered animals [4,5,6]. The second approach involves in vivo gene delivery targeted to postimplantation fetuses and requires surgical manipulation (also called intrauterine gene delivery or in utero gene delivery) in anesthetized mice. For example, an incision is made in pregnant mice, and genome-editing components are subsequently injected under microscopic observation using a glass needle, as depicted in Figure 1A,B. Unlike in the first approach, some restricted organs or tissues (such as the heart, brain, and lung) can be genome-edited here, which may frequently generate “mosaic” fetuses with genome-edited and unedited cells. A third approach involves directly introducing genome-editing components (i.e., a plasmid that confers expression of both Cas9 and gRNA complexed with a gene delivery reagent) to pregnant female animals at the mid-gestational stage via tail-vein injection. This method is called “transplacental gene delivery to acquire genome-edited fetuses (TPGD-GEF)” ([7], illustrated in Figure 1C). The reagents administered into the maternal bloodstream were transferred via the placenta to the fetuses, resulting in the generation of “mosaic” fetuses with some successfully genome-edited fetal cells (reviewed by Nakamura et al. [8]). A fourth approach requires facial or tail-vein injection of genome-editing reagents into newborn or adult mice or local administration to specific organs in adult mice (reviewed by Liu et al. [9] and Fajrial et al. [10]). This approach is called “somatic cell genome editing” [4] or “in vivo somatic cell gene editing” [11]. This approach also generates mosaic individuals, as do the second and third approaches. Even with the limitations of this approach, it is possible to cure abnormalities in specific organs by correction of mutant target genes or induction of gene mutations within target organs as an alternative to the transgenesis required for genome editing.

Therapeutic treatment in utero through genome editing offers several advantages over postnatal treatment, including delivery of a higher effective dose, generation of immune tolerance, and possible prevention of the phenotypic onset of genetic diseases in earlier stages of development. In utero gene therapy using genome-editing technology (as exemplified by CRISPR/Cas9 system) has shown promise in animal models. However, translational research from small animals to large animals and, subsequently, to humans will require extensive examinations of efficacy and maternal and fetal safety. Additional studies, including the determination of the optimal gestational age, delivery route, and social and ethical factors, are required prior to clinical translation.

The aim of this review is to describe the current state of the fetal-stage gene editing approaches (including the second and third approaches), which are now considered promising for fetal gene therapy. Here, we review the latest gene editing techniques used at the fetal stage, together with the possibilities and limitations of these technologies.

## 2. In Utero Gene Delivery

Most studies on gene delivery to egg cylinder (E7.5; E0 is defined as a day when copulation plug is observed after mating with males) or somite (E8.5) stage embryos have focused on the in vitro manipulation of the isolated embryos, except for the studies by Ngô-Muller and Muneoka [12], Sheehy et al. [13], and Endo et al. [14]. For example, Sheehy et al. [13] injected a 3-μL solution containing miR-452 antagomir, a specific inhibitor, to block the function of microRNA (miRNA)-452, using a 35-gauge needle into the space between the embryo and the YS through the decidua of an E8.5 embryo, under anesthesia (Figure 1A). After surgery, the treated embryos were allowed to develop to mid-gestational stages for sampling. In the samples analyzed, neural crest cell-specific reduction of miRNA, a small non-coding RNA, was observed, which was also associated with the generation of abnormal fetuses lacking their craniofacial cartilaginous structures. However, until now, there has been no successful report on in vivo genome editing in somite-stage embryos.

Experiments using in utero gene delivery methods are usually performed on mid- to late-gestational fetuses (from E9–18), probably because they are relatively easier to be identified by visual observation and also can be used in basic research on in utero gene therapy [15]. All these experiments were based on the opening of the abdominal portion of an anesthetized pregnant animal, exposure of uterine horns, and injection of a solution into a specific site of a fetus [16,17,18,19,20,21,22,23,24,25,26,27,28,29] or its surrounding or associated tissues, such as placenta [30,31,32], amniotic cavity [13,14,26,33,34,35], and the yolk sac (YS) [36], using a glass micropipette (shown in Figure 1B). The solution contains viral vectors (including recombinant adeno-associated viruses (rAAVs), lentiviruses, adenoviruses (Ad)), or nonviral vectors (including plasmid DNA). The injected site of nonviral naked plasmid DNA was subjected to in utero electroporation (EP) for harnessing DNA incorporation into cells of a specific fetal region.

After fetal gene delivery, the females were allowed to survive for a short period (2–4 days) for subsequent analysis or, in some cases, for the delivery of their pups.

### 2.1. Site-Directed in Utero Gene Delivery and Subsequent in Utero EP

Since the possibility of a genome-editing system that can be applied to the generation of genome-edited animals was shown in late 2013, an attempt to perform in utero gene delivery of genome-editing components has been made by several laboratories (Table 1). In 2014, Straub et al. [37] first performed the proof-of-principle demonstration of CRISPR/Cas9-mediated knock-down (KD) in neurons in vivo using in utero EP. They performed in utero EP of the head of E15 wild-type C57BL/6J fetuses after injecting 1 µL of DNA mixture into the left hemisphere using a 50-µm-diameter pipette. The DNA mixture contained the CRISPR-construct (targeted to glutamate ionotropic receptor N-methyl-D-aspartate [NMDA] type subunit 1 [*Grin1*] in a sparse population of mouse pyramidal neurons) and soluble green fluorescent protein (GFP) (10:1) + 0.005% fast green (for visualization of a solution injected). When the transfected pups were checked for synaptic current mediated by NMDA-type glutamate receptors 14–20 days following birth, manipulated cells (comprised of genetically mosaic cells) lacked it, being consistent with Grin1 loss-of-function phenotype obtained using *Grin1* KD mouse (GluN1KD) [38]. This study suggests that in utero CRISPR/Cas9-mediated KD was useful for studying the function of specific proteins in neuronal circuits. Similar results were also obtained by Shinmyo and Tanaka [39], who injected pX330 plasmids expressing humanized Cas9 and single-guide RNAs against the special AT-rich sequence-binding protein 2 (*Satb2*) gene into the developing mouse brain [at E15.5] and, subsequently, performed in utero EP. Kalebic et al. [40] performed in utero EP of a single plasmid encoding *Cas9* and an appropriate gRNA into the embryonic neocortex.

Uemura et al. [41] employed in utero EP to enable CRISPR/Cas9-mediated gene KI of enhanced GFP (EGFP) coding sequence at the site immediately after the first ATG codon of the β-actin gene in fetal neurons. Consequently, EGFP-tagged β-actin protein expression was discernible in the cortical layer 2/3 pyramidal neurons. According to Uemura et al. [42], this epitope tagging-based approach is particularly useful for elucidating various endogenous protein localization in neurons without altering neuronal and synaptic functions.

**Table 1:** Summary of the characteristics of genome-editing experiments targeting developing murine fetuses reported to date.

Type of Method	Genome-Editing Tool (Mode for Gene Modification)	Outcome	Target Gene	Data from
In utero gene delivery and subsequent in vivo electroporation (EP) (all-in-one plasmid)	CRISPR/Cas9 (indels)	Performed at E15 of pregnancy; first successful knock-down (KD) in pyramidal neurons in vivo; manipulated cells lacked synaptic current mediated by NMDA-type glutamate receptors	*Grin1*	[37]
In utero gene delivery and subsequent in vivo EP (all-in-one plasmid)	CRISPR/Cas9 (indels)	Induced abnormalities in axonal projection patterns, which is consistent with the phenotypes previously observed in *Satb2* mutant mice	*Satb2*	[39]
In utero gene delivery and subsequent in vivo EP [all-in-one plasmid or ribonucleoprotein (RNP)]	CRISPR/Cas9 (indels)	Successful disruption of the expression of green fluorescent protein (*GFP*) or endogenous eomesodermin (*Eomes*)/T-box brain protein 2 (*Tbr2*), a gene fundamental for neocortical neurogenesis	*GFP* *Eomes/* *Tbr2*	[40]
In utero gene delivery and subsequent in vivo EP (all-in-one plasmid + donor plasmid DNA)	CRISPR/Cas9 (KI)	Successful knock-in (KI) of *EGFP* fragment into the *β-actin* locus and enhanced GFP (EGFP)-tagged β-actin protein in cortical layer 2/3 pyramidal neurons; provide a useful tool to detect the localization of various endogenous proteins in neurons	*β-actin*	[41]
In utero gene delivery (*Cas9*-expressing plasmid + gRNA-expressing plasmids + donor plasmid DNA) and subsequent in vivo EP	CRISPR/Cas9 (KI)	Successful de novo targeted KI of *EGFP* sequence into the target locus through in utero EP into the mammalian brain	*βIII-tubulin (Tubb3)*	[43]
In utero delivery of Ad containing base editor 3 (BE3) and gRNA	CRISPR/BE3-based gene correction	Viral vector–mediated delivery of CRISPR/Cas9 or BE3 through in utero gene delivery through vitelline vein injection of E16 fetus was performed to pursue therapeutic modification of proprotein convertase subtilisin/kexin type 9 (*Pcsk9*) or hydroxyphenylpyruvate dioxygenase (*Hpd*) in wild-type mice or a murine model of hereditary tyrosinemia type 1 (HT1), respectively; long-term postnatal persistence of edited cells were observed in both models, with reduction of plasma PCSK9 and cholesterol levels following in utero *Pcsk9* targeting and rescue of the lethal phenotype of HT1 following in utero *Hpd* targeting.	*Pcsk9* *Hpd*	[44]
In utero delivery of nanoparticle (NP) carrying peptide nucleic acids (PNAs) + donor DNA	PNA and DNA oligomers-based KI	Intravenous (via the vitelline vein) NP delivery of PNAs and single-stranded DNA (ssDNA) to mouse fetuses (at E15 to E16) harboring mutations in the human β-globin gene, which are recognized as a model for human β-thalassemia, resulted in site-specific genome-editing of fetal liver cells, leading to phenotypic rescue of thalassemia (severe anemia) before birth	*β-globin*	[45]
In utero delivery of adenoviruses (Ad) carrying Cas9, gRNA or donor DNA	CRISPR/Cas9 (KI)	In utero delivery of Ad carrying CRISPR reagents into the amniotic cavity of a fetus (at E16) resulted in transduction of alveolar epithelial cells of fetal lung, extensive pulmonary gene editing, and, finally, rescued a perinatal lethal phenotype in the surfactant protein C (Sftpc)^I73T^ mice, a model for monogenic lung disease	*Sftpc*	[46]
Transplacental gene delivery to acquire genome-edited fetuses (TGPD-GEF) (all-in-one plasmid)	CRISPR/Cas9 (indels)	TPGD-GEF causes indel mutations in embryonic cardiomyocytes of mid-gestational murine fetuses	*EGFP*	[7]
In utero delivery of CRISPR-AAV9	CRISPR/Cas9 (indels)	Injection of recombinant adeno-associated viruses (rAAVs) carrying short Cas9 variant and gRNA into fetal brain (at E15) of mouse model of Angelman syndrome (AS) resulted in successful unsilencing of paternal ubiquitin–protein ligase E3A (*Ube3a*) throughout the brain for at least 17 months and rescued abnormal phenotypes associated with AS mice	*Snord115 on* *UBE3A*	[47]
TPGD-GEF (all-in-one plasmid)	CRISPR/Cas9 (indels)	Hydrodynamics-based gene delivery (HGD) into pregnant female mice at E9.5 resulted in mosaic indel mutations in myosin heavy chain α (*MHCα*) with an efficiency of 40%	*MHCα*	[48]
In utero gene delivery and subsequent in vivo EP (all-in-one plasmid)	CRISPR/Cas9 (KI)	Development of a novel method, called “Targeted KI with Two”, for precise genome-editing-based tagging in mouse primary cultured neurons with efficiencies up to 42%; when injection of CRISPR reagents into the ateral ventricle of fetal brain (at E15) and subsequent in utero EP was performed, expression of donor DNA was observed along the dendrites of layer 2/3 pyramidal neurons and its expression lasted more than 1 year, indicating the long-term stability of the KI tag	*Gria2*	[49]
In utero delivery of rAAV9 carrying adenine base editor (ABE)	ABE system	In utero injection of rAAV9 carrying the ABE targeting the Idua G→A (W392X) mutation in the mucopolysaccharidosis type I (MPS-IH) mouse, corresponding to the common IDUA G→A (W402X) mutation in MPS-IH patients, was performed via vitelline vein of a fetus at E15; which resulted in long-term W392X correction in hepatocytes and cardiomyocytes and low-level editing in the brain, highlighting the potential of this approach for MPS-IH and other genetic diseases	*Idua*	[50]
In utero delivery of CRISPR-AAV9-PHP.eB carrying gRNA	CRISPR/Cas9 (indels)	In utero injection of rAAV9-PHP.eB (AAV9-based mutant capsid that is highly efficient in transducing the central nervous system of adult mice) carrying gRNA to the lateral ventricles of fetal mouse brain (at E15) of *Cas9* transgenic (Tg) mice resulted in widespread gene KO; suggesting a useful platform for studying brain development and devising genetic intervention for severe developmental diseases	*PogZ* *Depdc5*	[51]
In utero delivery of rAAV9 carrying ABE	ABEmax-NG system (ABEmax combined with SpCas9-NG (capable of recognizing NG rather than NGG)	In utero injection of rAAV9 carrying the ABEmax-NG into the vitelline vein of E16 fetus resulted in about 25.3% correction of the pathogenic hypertrophic cardiomyopathy (HCM) mutation (R404Q/+ mutation; Myh6 c.1211C > T) in a mouse model of HCM, and reduced expression of mutant RNA, suggesting that ABEmax-NG has the potential to correct the HCM mutation in vivo	*Myh6*	[52]

### 2.2. Intraamniotic Injection of Viral Vectors Carrying Genome-Editing Components

Rossidis et al. [44] demonstrated proof that the viral vector-mediated delivery of CRISPR/Cas9 or base editing could be applied in utero to explore the therapeutic possibilities to cure lethal peri- and neonatal diseases. They first injected adenoviral particles containing the CRISPR-base editing system (targeting proprotein convertase subtilisin/kexin type 9 (*Pcsk9*), which regulates low-density lipoprotein-receptor proteins) into the vitelline vein of wild-type murine fetuses at E16. These particles are incorporated into the blood from the YS. Notably, in the previously established *Pcsk9* KO mice, plasma cholesterol levels and risk of coronary heart disease were reduced [53]. In utero administration of adenoviral particles generated approximately 10% base-edited alleles, sufficient to lower cholesterol levels. Moreover, a very low rate of indels was observed. Next, Rossidis et al. [44] demonstrated the possibility of in utero base editing to rescue a neonatally lethal disease using a mouse model for hereditary tyrosinemia type 1 (HT1), a metabolic liver disease caused by a mutation in the fumarylacetoacetate hydrolase (*Fah*) gene. This mutation blocks the tyrosine catabolic pathway, leading to toxic metabolite accumulation and death within the first months after birth. *Fah* KO mice exhibit neonatal lethality within 20 days after birth [54]. In utero, base editing targeting hydroxyphenylpyruvate dioxygenase (*Hpd*) was performed into fetuses derived from *Fah* KO mice at E16. Consequently, it was shown that the lethal phenotype of *Fah* KO mice could be successfully rescued because the born mice survived up to at least three months.

Gene editing, such as CRISPR/Cas9, is now an efficient tool for treating genetic disorders by correcting disease-causing mutations (reviewed by Harrison et al. [1]). To correct disease-causing mutations, HDR-based genome editing has been frequently employed; however, its efficiency remains low. To circumvent this problem, CRISPR/Cas9-based base editing technology was recently developed (reviewed by Antoniou et al. [42]). This system employs an enzymatically inactive-Cas9 fused to a cytidine deaminase, called a base-editing complex, and can change C-G base pairs into T-A. The base-editing complex can be targeted to a specific locus in combination with the use of gRNA. It edits the sequence within a window of approximately five nucleotides without inducing DSB. Notably, this phenomenon is HDR-independent.

### 2.3. In Utero Injection of Viral Vectors Carrying Genome-Editing Components into the Brain

Angelman syndrome (AS) is associated with several abnormal phenotypes, including severe developmental delays, seizures, impaired speech, and, often, autism. Patients with this disease have a mutation or deletion of the maternally inherited ubiquitin–protein ligase E3A (*UBE3A*) allele. In neurons, the paternally inherited *UBE3A* allele is silenced in *cis* by a long non-coding RNA called *UBE3A-ATS* (*UBE3A* antisense transcript). To cure AS-related neurological abnormality, it is necessary to modulate signals between neurons. Wolter et al. [47] first evaluated the extent to which directed Cas9 targeting of small nucleolar RNA, C/D box 115 (*SNORD115*) genes, which are small nucleolar RNAs (snoRNAs) clustered in the 3’ region of *UBE3A-ATS*, can unsilence paternal *UBE3A* in primary human neural progenitor-derived neurons. In this case, a single nucleotide variant in *UBE3A* exon 5 was assessed by qRT-PCR using single-nucleotide variant-specific qRT-PCR probes to quantify maternal and paternal allelic expression of *UBE3A*. The presumed maternal *UBE3A* allele was predominantly expressed (nine-fold higher than the paternal allele) in differentiated neurons, coinciding with the emergence of paternal *UBE3A-ATS* expression. When these human neural progenitor-derived neurons were transduced with a lentiviral vector carrying the *Cas9* gene and gRNA, fluorescence-activated cell sorting (FACS)-sorted Cas9^+^ cells exhibited increased expression of the paternal *UBE3A* allele, comparable to maternal *UBE3A* levels, suggesting the fidelity of this approach.

Wolter et al. [47] injected an rAAV9 vector carrying a short Cas9 variant (Staphylococcus aureus Cas9 (SaCas9)) and a gRNA targeting *Snord115* into the fetal brains (intracerebroventricularly, bilaterally at E15) of AS model mice to restore expression of paternally inherited *Ube3a*. This early treatment unsilenced paternal *Ube3a* throughout the brain for at least 17 months, rescuing anatomical and behavioral phenotypes in AS mice. These studies show that targeted genomic integration of a gene therapy vector can restore paternally inherited *UBE3A* functions throughout life. The strategy employed by Wolter et al. [47] may provide a way toward disease-modifying treatments for other neurodevelopmental disorders caused by mutations in single genes.

### 2.4. Intraamniotic Injection of Non-Viral DNA Encapsulated with Lipids

Ricciardi et al. [45] performed intraamniotic or intravenous (via the vitelline vein) administration of biodegradable NP (300 or 400 mg kg^−1^ per fetus based on an average E15 fetal weight of 0.45 g) derived from poly (lactic-co-glycolic acid) containing triplex-forming peptide nucleic acids (PNAs) and donor single-stranded DNA (ssDNA) into mouse fetuses (at E15 to E16) harboring mutations in the human β-globin gene (a recognized model for human β-thalassemia). They observed that intravitelline vein delivery of NP resulted in widespread particle distribution throughout the fetus at both E15 and E16, with the most abundant NP accumulation in the fetal liver, where rapid hematopoietic stem cell expansion occurs. When treated mice were examined 10 weeks after intravenous injection at E15 for the possible elevation of hemoglobin concentrations at both doses, fetuses treated with NP were found to develop into adult mice with significantly higher levels of blood hemoglobin than untreated β-thalassemic mice. A higher dose of NP-carrying PNAs and ssDNA resulted in a greater increase in hemoglobin concentration, yielding values in the wild-type range. Deep sequencing analysis for β-globin gene editing in bone marrow cells isolated from E18 fetuses revealed 8.81% editing. A droplet digital PCR assay also confirmed an average of ~6% editing, consistent with the deep sequencing data. The resulting offspring exhibited a successfully rescued phenotype, as exemplified by a reduced number of reticulocytes, the disappearance of splenomegaly, and longer survival. Ricciardi et al. [45] suggested that in utero gene editing has the potential to be safe and produce a clinical response substantial enough to reduce β-thalassemia-associated morbidity and mortality.

**Figure 2:**
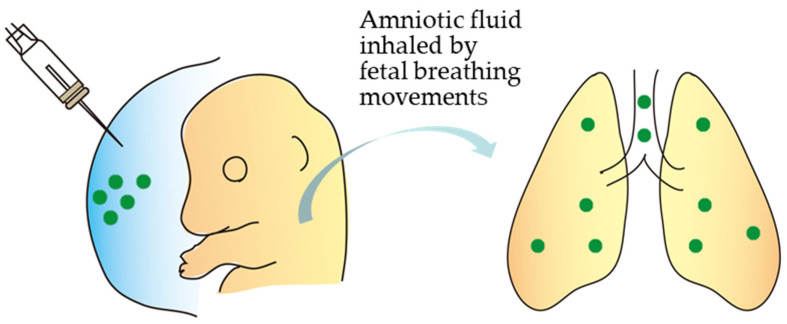
Substances introduced via intraamniotic injection can be taken up by a fetus (at E16). According to Ricciardi et al. [45], intraamniotic injection at E15 did not lead to any detectable accumulation of materials injected within the fetus. However, injection at E16—the expected time of onset of pronounced fetal breathing and swallowing—resulted in material accumulation in the fetal lung and gut. This figure was drawn in-house and reproduced based on Alapati et al. [46].

Alapati et al. [46] demonstrated that CRISPR/Cas9 technology could rescue a lethal perinatal monogenic lung disease. A CRISPR/Cas9 vector was subjected to in utero intraamniotic delivery at E16 of pregnancy in fetuses carrying a mutation (called *SftpcI73T*) in the lung-disease-causing *Sftpc* gene. Amniotic fluid was then inhaled by fetal breathing movements, as schematically shown in Figure 2. *SftpcI73T* is a mutated version of the pulmonary-associated protein C gene encoding a protein that helps prevent the lung from collapsing when emptied. Embryonic expression of *SftpcI73T* causes severely diffused parenchymal lung damage, leading to the early death of affected individuals. Gene editing successfully targeted the epithelial lining cells of the lungs of 20% of the mice born. When gene-edited cells were assessed using flow cytometry and immunohistochemistry for possible persistence of the lung-disease-causing gene, the percentage of gene-edited lung epithelial cells remained unaltered after six months. Notably, no genetic alteration was detected in the germ cells, suggesting the safety of this technology. This study demonstrates the feasibility of this novel fetal gene-editing method for curing human monogenic disorders.

## 3. TPGD-GEF Technique

In the postimplantation stages, the embryo is surrounded by maternal tissues and becomes more dependent on maternal nutrition as it grows. During placenta formation after embryo implantation, the embryo is separated by a specific but still not fully mature barrier from the mother’s blood circulation. When the placenta is completely formed (which is considered at E14.5 [55]), it functions as the maternal–fetal barrier (also called placental barrier or biological barrier between the mother and fetus) mediating the maternal–fetal transfer of a large variety of substances (such as carbohydrates, fats, dietary fiber, minerals, protein, vitamins, and water). In some cases, cells, viruses (i.e., human immunodeficiency virus (HIV), rubella virus, human papillomavirus, and hepatitis C), and nucleic acids such as miRNA present in maternal circulation can be transferred to the fetus (reviewed by Tetro et al. [56] and Figueroa-Espada et al. [57]). Notably, Tüzel-Kox et al. [58] demonstrated that liposomes injected intravenously into pregnant rats could be trapped in the placenta. The entrapped materials are then transported to the fetus as free degraded molecules. These results suggest that nucleic acids, such as plasmid DNA encapsulated with liposomes, may be transferred via the maternal–fetal barrier to the fetuses when intravenously injected into a pregnant mouse.

Tsukamoto et al. [59] first showed evidence of transplacental delivery of nucleic acids to fetuses at early-to-mid gestational stages (E9 to 15) when a single tail-vein injection of a plasmid carrying the *lacZ* gene (encoding β-galactosidase) complexed with Lipofectamine (5-carboxyspermylglycine dioctadecylamide (DOGS)) was administered. Notably, fetuses treated at E9 contained at least 40 times more plasmid DNA than those treated at E12 or E15. No plasmid DNA was detected in fetuses treated at E3 or E6. Furthermore, fetuses recovered after transplacental gene delivery (TPGD) exhibited blue deposition due to gene expression of *lacZ* derived from the introduced plasmid when stained in the presence of X-Gal, a substrate for β-galactosidase. This means that plasmid DNA injected into the tail veins of pregnant female mice can be successfully delivered through the placental interface to the developing fetuses, and fetal cells are effectively transfected with the introduced DNA. Since the report by Tsukamoto et al. [59], several researchers have demonstrated the feasibility of this technology (TPGD) for RNA interference (RNAi)-based suppression of a target gene using short hairpin RNA (or small interfering RNA) and miRNA [15,60,61] and genetic immunization of fetuses [62]. The feasibility of using liposomal reagents and other reagents, such as cationic tetraamino fullerene, to deliver plasmid DNA through TPGD is also shown by several groups [63,64,65,66]. Since this technique does not require surgery, such as in utero gene delivery experiments, and, therefore, is noninvasive toward pregnant females, it can be a useful tool for studying the effects of genes on embryonic development (reviewed by Nakamura et al. [8]).

**Figure 3:**
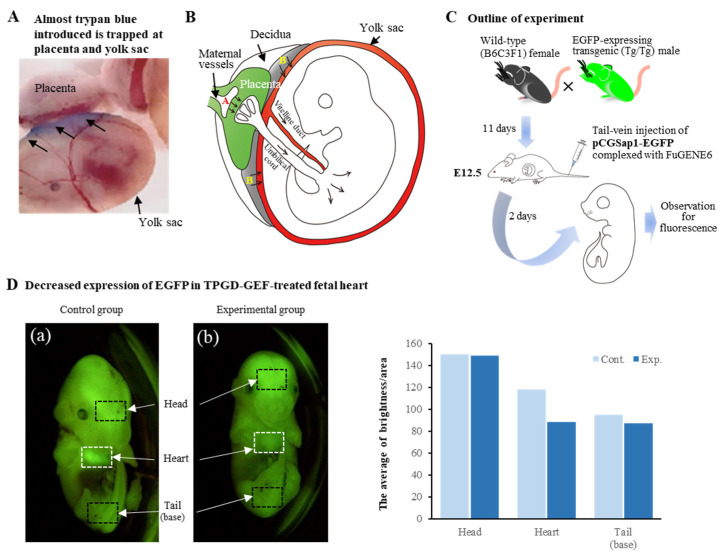
Transplacental gene delivery (TPGD) at E12.5. (**A**) Localization of trypan blue in the isolated fetus with placenta and yolk sac (YS) one day after tail-vein injection into a pregnant female at E12.5. Notably, almost trypan blue was trapped in the placenta and YS (arrows). The photo is based on the picture from Kikuchi et al. [64] published by Nature Publishing group, 2002. (**B**) Hypothetical mechanism of TPGD as suggested by Kikuchi et al. [64]. Following TPGD on E12.5, when placental circulation is established, intravenously injected plasmid DNA/lipid complexes may be transferred from maternal blood to the fetus via at least two routes. Flow via the placenta to the embryo is indicated by the arrows in area A; injected plasmid DNA is transferred beyond the blood–placenta barrier and enters the umbilical cord. Flow from the decidua to the YS is indicated by the arrows in area B; some DNA becomes trapped in YS and is transferred to the embryo after the establishment of functional placental circulation. This figure was drawn in-house and reproduced with permission from Nakamura et al. [8] published by MDPI, 2019. (**C**) Schematic representation of the experimental outline of TPGD-GEF. At E12.5, a solution containing plasmid DNA complexed with gene delivery reagent (i.e., FuGENE6) was intravenously administered to the pregnant female mice. Two days after the in vivo transfection, fetuses were dissected to check the expression of the introduced DNA. (**D**) Decreased expression of EGFP-derived fluorescence in the TPGD-GEF-treated fetus. In the intact control fetus (Control group), the heart exhibited strong fluorescence, whereas some TPGD-GEF-treated fetuses exhibited reduced fluorescence in their hearts (Experimental group). The number of pixels of “luminance” in each area is analyzed in the software’s histogram function (Adobe Photoshop Elements 2018) and plotted as a graph (right panel). The areas analyzed are the three sites (head, heart, and base of the tail) shown in the boxes of the figure. Notably, fluorescence in the genome-edited fetal heart area was greatly reduced compared to that in unedited fetuses, suggesting extensive genome editing in the hearts of genome-edited fetuses. The figures in (**C**,**D**) were drawn in-house and reproduced with permission from Nakamura et al. [7], published by Wiley, 2019.

We assessed the possible mechanism of TPGD in pregnant females at E12.5 to 13.5 by intravenous injection of trypan blue, a vital dye for monitoring the fate of the injected substance [64]. When fetuses with YS and placenta were dissected one day after dye injection and inspected for possible transplacental delivery of dye into the fetuses, no appreciable presence of dye was discernible. Almost all the dye was trapped in the placenta and YS (arrows in Figure 3A). However, the presence of exogenous DNA was still discernible in some of the fetuses when a genomic PCR was carried out using the isolated fetuses two days after TPGD with a *Cre* expression plasmid DNA complexed with FuGENE6, a lipid specified to facilitate DNA delivery [64]. Furthermore, staining of the isolated fetuses in the presence of X-Gal revealed predominant staining in the fetal heart and weak staining in the head and peripheral portion of vertebrae.

According to Kikuchi et al. [64] and Nakamura et al. [8], in the early stages of postimplantation (E5.5 to E9.5), substances, including DNA/lipid complexes in maternal blood, are taken up by the visceral endoderm (VE) or YS and then transported to embryos by diffusion or vitelline circulation. During placental maturation, it may become a major tissue for controlling nutrient transfer in maternal blood (Figure 3B). Most DNA/lipid complexes may be trapped in the VE/YS/placenta, and small amounts may be taken up and transported to the fetuses. Indeed, successful TPGD has been reported at E12.5 and E11.5 for delivering AAV particles [67] and plasmid DNA/liposome complexes [63], respectively. On the other hand, other groups successfully performed TPGD at E9.5 and E6.5 for delivering RNAi [15] and plasmid DNA/liposome complexes [59], respectively. From these experiments, we concluded that the exogenous DNA, such as plasmid intravenously administrated into a pregnant female at early-to-mid gestational stages, can be transferred via the placenta into fetuses. However, it is present transiently in fetuses, and the transfection rate varies among pregnant females.

As mentioned previously, CRISPR/Cas9-based genome-editing system uses only two components, namely, Cas9 endonuclease and gRNA. Transient expression of these components at the transfected cells or tissues is enough to induce indel-based mutations at a target locus. As a proof-of-principle experiment showing that TPGD can meet such demand for producing genome-edited fetuses, we first performed TPGD using an all-in-one type of plasmid. This plasmid, pCGSap1-*EGFP*, confers simultaneous Cas9 and gRNA expression targeted to *EGFP* cDNA upon transfection [7]. A solution containing pCGSap1-*EGFP* complexed with FuGENE6 was intravenously injected into the tail vein of pregnant wild-type female mice that had already been mated with male Tg mice carrying *EGFP* transgenes in a homozygous (Tg/Tg) state at E12.5 (see Figure 3C). Without CRISPR/Cas9 system application, all the fetuses should express *EGFP* systemically because they carry the transgenes in a heterozygous (Tg/+) state. However, TPGD-based delivery of CRISPR reagents targeted to *EGFP* will likely reduce EGFP fluorescence levels in these fluorescent fetuses due to genome editing in one allele having the chromosomally integrated *EGFP* transgenes. When fluorescence was inspected for fetuses isolated two days after TPGD, three of 24 fetuses exhibited reduced fluorescence in their heart (a vs. b in Figure 3D), consistent with the previous finding that the fetal heart is an organ preferentially transfected with TPGD [64]. Molecular biological analysis of these isolated fetuses demonstrated the presence of the transgene construct (*Cas9* gene) and indels at the target *EGFP* sequence. Notably, these fetuses showing reduced fluorescence comprised genome-edited and unedited cells as mosaic mutations. These results suggest that this TPGD-based genome editing, called TPGD-GEF, is effective in causing mutations at a target locus in a specific part (embryonic heart, in this case) of a fetus. It can potentially produce cardiovascular disease models and aid in basic research on fetal gene therapy for congenital heart diseases.

We further extended the applicability of TPGD-GEF to show that the endogenous gene can be disrupted by this technique [48]. We first prepared an all-in-one type of plasmid, pCGSap1-*MHC*, which confers simultaneous expression of *Cas9* and gRNA targeted to endogenous myosin heavy chain α (*MHCα*) gene upon transfection. KO *MHCα* mice exhibited heart failure due to acute cardiac hypertrophy [68]. A solution containing pCGSap1-*MHC* encapsulated with FuGENE6 was intravenously administered through the HGD approach to pregnant females at E9.5 or E12.5. When fetuses were inspected two days after TPGD-GEF, only one female was confirmed to have genome-edited fetuses out of four females treated at E9.5 with a ratio of 40%. However, none of the genome-edited fetuses were obtained from the other two pregnant females (22 fetuses tested). Notably, none of the genome-edited fetuses were obtained from three pregnant (E12.5) females (31 fetuses tested). Molecular analysis revealed that all genome-edited fetuses recovered comprised a mixture of genome- and non-genome-edited cells. This mosaicism was found in the fetal heart and other organs. These findings suggest the variability and feasibility of TPGD-GEF coupled with hydrodynamics-based gene delivery (HGD) in E9.5 fetuses for the possible production of individuals with heart failure as a disease model.

## 4. Limitations of and Possibilities for In Utero Genome Editing and TPGD-GEF

Despite the potential risk of frequent embryonic lethality, treatment in utero, also called in utero gene therapy, fetal gene therapy, or prenatal gene editing, offers several distinct advantages over postnatal treatment. Small fetus size allows the delivery of a higher effective dose of the gene therapy. Immune tolerance can be stimulated, and the phenotypic onset of genetic diseases that manifest at perinatal stages can be prevented. Table 2 summarizes previous genome-editing applications targeting developing murine fetuses. This table describes several parameters of each procedure, such as ease of the procedure, tissue type mainly transfected, efficacy, safety, and cost.

The past experiments using in utero genome-editing approach and TPGD-GEF have demonstrated the feasibility of CRISPR/Cas9-based genome editing in mice. Most studies rely on physical approaches, including glass micropipette-aided injection, EP, tail-vein injection, and HGD. In utero, genome editing is always associated with a surgical treatment requiring a microscopic-guided injection of the gene-editing cargo into the target cells. However, the latter is technically difficult to perform and often a cause for fetal death due to mechanical damages. EP after in utero gene delivery is also one of the risk factors leading to fetal death, which, therefore, requires careful optimization of EP conditions. HGD requires introducing a large amount of fluid at once, which often causes physiological damage to the liver and other organs. Furthermore, the reagents used for in vivo genome editing of fetuses are viral vectors (including rAAVs, Ad, and retroviral vectors) as well as nonviral vectors (including plasmid DNA). Safety is the primary concern with currently used viral vectors, especially Ad, which often elicits a potential immunological response when repeated infection trials are attempted [69].

Overall, nonviral nano-vectors, also called NP, can exhibit profound advantages over the abovementioned physical approaches and virus-based delivery. Particularly, tail-vein injection of nonviral vectors complexed with DNA delivery reagents, such as liposomes, would be an ideal approach to achieve genome editing in fetuses because it is safer and relatively noninvasive than in utero genome editing. However, low genome-editing frequency remains an issue to be dissolved in the future.

To date, several types of nano-vectors, including lipids (such as 1,2-dioleoyl-sn-glycero-3-phosphoethanolamine [DOPE] and cholesterol), polymers (such as polyethyleneimine, poly-L-lysine, and chitosan), inorganic chemicals (such as cationic arginine gold nanoparticles and CRISPR-Gold), and exosomes (such as exosome-liposome hybrid and engineered exosomes: CD9-HuR exosomes and NanoMEDIC) have been developed [70,71,72]. Since these nano-vectors can prevent the degradation of the CRISPR/Cas9 genome-edition system and directly enter into the nucleus to perform genome editing in vivo, they can be ideal delivery platforms for the CRISPR/Cas9 system, enabling effective ex vivo or in vivo transfection of CRISPR reagents encoding DNA, RNA, or RNP in a highly efficient and safe way [73,74,75]. For example, Wei et al. [76] encapsulated RNPs with cationic lipids composed of 1,2-dioleoyl-3-(trimethylammonium) propane as permanent cationic supplements, DOPE as a helper lipid, and cholesterol as a sterol, postmodified with polyethylene glycol phospholipid as PEGylated lipids to yield nanoparticles with retained activity and redirect DNA editing to the target tissues with decreased clearance and immunogenicity. They demonstrated that low-dose intravenous injections could effectively target specific tissues, including the sphincter muscles, brain, liver, and lungs. Aside from the study of Wei et al. [76], there have been many successful reports for in vivo genome editing using lipids [77,78,79,80], polymers [81,82,83,84,85,86,87], inorganic nanomaterials [88,89,90,91,92,93], and exosomes [94,95,96,97].

These nano-vectors can be easily scaled up and modified chemically. They are cost-effective, have large packaging capacity, and lower immunogenicity, which will match the demand for in utero and tail-vein injection-based gene delivery to fetuses. Notably, Thermo Fisher Scientific has formulated a lipid-based chemical transfection reagent optimized to deliver Cas9 RNP complexes [98].

## 5. Conclusions

For in utero gene delivery to early postimplantation embryos, manipulation of embryos (at E6–8) has been long thought to be difficult in vivo because they are surrounded by decidua. Fortunately, Sheehy et al. [13] have suggested that in vivo gene delivery to embryos at these stages is indeed possible via a glass capillary-based injection of a nucleic acid-containing solution under a dissecting microscope. Although there is no report on successful genome editing at this stage, future work will soon dissolve this issue.

For in utero gene delivery to mid-to-late postimplantation fetuses, successful genome editing has already been reported by several laboratories using various methods, such as in utero EP, intraamniotic injection, and TPGD-GEF. The substances employed are genome-editing reagents (*Cas9* mRNA + gRNA or Cas9 RNP; in some cases, the donor DNA is included), nano-vectors, such as lipid-based nanoparticles, and viral or non-viral vectors carrying genome-editing components. Surgery and EP apparatus (for in utero EP), which are often invasive to the survival of fetuses, are always required for in utero genome editing. Ad vector introduction often causes immunogenic responses. Tail-vein injection, a noninvasive approach for inducing genome editing at a target locus, is required for TPGD-GEF. However, the efficiency is low with frequent mosaic mutations. To date, nanoparticle-based delivery of genome-editing reagents provides higher genome-editing efficiency than that achieved by the previous approach based on the commercial lipid (liposome)-based reagents. In this context, tail-vein-mediated delivery of genome-editing reagents complexed with nanoparticles may be one of the ideal approaches for inducing targeted mutations in a non-invasive manner. Combinational use of these particles with TPGD-GEF may overcome the disadvantages associated with the low efficiency of genome editing.

In utero genome editing is now a promising tool for curing embryonic lethality of fetuses with inherited genetic disorders because researchers from the Children’s Hospital of Philadelphia (CHOP) and the Perelman School of Medicine at the University of Pennsylvania succeeded in rescuing fetuses that were prone to prenatal death [44,46,50]. Although there are still many hurdles to applying this technology to clinical therapeutics, the ongoing advancement of technologies will overcome these.

## Figures and Tables

**Table 2:** Summary of genome-editing applications targeting developing murine fetuses.

Procedure	Ease of Procedure	Tissue Type	Efficacy	Safety	Cost	Data from
In utero injection of plasmid DNA and subsequent in vivo EP	Relatively easy but accompanied with surgical procedure	Brain	Depending on the technique of the researchers	Risk of embryonic loss and tissue damage during surgery	Requires electroporator and microinjector device (in some cases)	[37,39,40,41,43,49]
In utero injection of Ad containing BE3-based components	Relatively easy but accompanied with surgical procedure	Lung, intestine	High	Risk of embryonic loss and tissue damage during surgery and also for immunologic response	Requires microinjector device (in some cases)	[44,46]
In utero injection of NP containing PNAs and donor DNA	Relatively easy but accompanied with surgical procedure	Whole fetus (especially accumulated abundantly in fetal liver)	Relatively high	Risk of embryonic loss and tissue damage during surgery	Requires microinjector device (in some cases)	[45]
TPGD-GEF using liposome-encapsulated plasmid DNA	Easy	Whole fetus (especially accumulated abundantly in fetal heart)	Low	Very low risk for both mother and fetuses (however, HGD, poses risk of damage in both mother and fetus)	Requires only tail-injection technique	[7,48]
In utero injection of rAAVs containing CRISPR/Cas9 components	Relatively easy but accompanied with surgical procedure	Brain	High	Risk for embryonic loss and tissue damage during surgery; risk of immune response	Requires microinjector device (in some cases)	[47,49,51]
In utero injection of rAAVs containing ABE	Relatively easy but accompanied with surgical procedure	Whole fetus (especially abundantly accumulated in fetal liver)	High	Risk for embryonic loss and tissue damage during surgery; risk of immune response	Requires microinjector device (in some cases)	[50,52]

## Data Availability

Not applicable.

## Abbreviations

AAV	Adeno-associated viruses
ABE	Adenine base editor
Ad	Adenoviruses
AS	Angelman syndrome
BE3	Base editor 3
Cas9	CRISPR-associated protein 9
CRISPR	Clustered regularly interspaced short palindromic repeats
DOGS	5-Carboxyspermylglycine dioctadecylamide
DOPE	1,2-Dioleoyl-sn-glycero-3-phosphoethanolamine
dsDNA	Double-stranded DNA
DSB	Double-strand break
E	Embryonic day
EGFP	Enhanced green fluorescent protein
*Eomes*	Eomesodermin
EP	Electroporation
*Fah*	Fumarylacetoacetate hydrolase
FACS	Fluorescence-activated cell sorting
*GFP*	Green fluorescent protein
*Gria2*	Glutamate ionotropic receptor AMPA type subunit 2
*Grin1*	Glutamate ionotropic receptor NMDA type subunit 1
gRNA	Guide RNA
HCM	Hypertrophic cardiomyopathy
HDR	Homology directed repair
HGD	Hydrodynamics-based gene delivery
HIV	Human immunodeficiency virus
*Hpd*	Hydroxyphenylpyruvate dioxygenase
HT1	Hereditary tyrosinemia type 1
*Idua*	α-L-iduronidase
KD	Knock-down
KI	Knock-in
KO	Knockout
*MHCα*	Myosin heavy chain α
miRNA	MicroRNA
MPS-IH	Mucopolysaccharidosis type I
*Myh6*	Myosin heavy chain 6
NHEJ	Non-homologous end joining
NMDA	N-methyl-D-aspartate
NP	Nanoparticle
*Pcsk9*	Proprotein convertase subtilisin/kexin type 9
PNAs	Peptide nucleic acids
*PogZ*	*Pogo transposable element derived with ZNF domain*
RNAi	RNA interference
RNP	Ribonucleoprotein
rAAVs	Recombinant adeno-associated viruses
SaCas9	Staphylococcus aureus Cas9
*Satb2*	Special AT-rich sequence-binding protein 2
*Sftpc*	Surfactant protein C
ssDNA	Single-stranded DNA
*Snord115*	Small nucleolar RNA, C/D box 115
snoRNAs	Small nucleolar RNAs
*Tbr2*	T-box brain protein 2
Tg	Transgenic
TPGD	Transplacental gene delivery
TPGD-GEF	Transplacental gene delivery to acquire genome-edited fetuses
*UBE3A*	Ubiquitin–protein ligase E3A
*UBE3A-ATS*	*UBE3A* antisense transcript
VE	Visceral endoderm
YS	Yolk sac
